# Establishing a novel colorectal cancer predictive model based on unique gut microbial single nucleotide variant markers

**DOI:** 10.1080/19490976.2020.1869505

**Published:** 2021-01-11

**Authors:** Chenchen Ma, Kaining Chen, Yuanyuan Wang, Chaoping Cen, Qixiao Zhai, Jiachao Zhang

**Affiliations:** a College of Food Science and Engineering, Hainan University, Haikou, Hainan, P. R. China; b Key Laboratory of Food Nutrition and Functional Food of HainanProvince, Hainan University, Haikou, Hainan, China; c Hainan General Hospital, Hainan Affiliated Hospital of Hainan Medical University, Haikou, P. R. China; d State Key Laboratory of Food Science and Technology, Jiangnan University, Wuxi, Jiangsu, P. R China; e School of Food Science and Technology, Jiangnan University, Wuxi, Jiangsu, China

**Keywords:** Metagenome, colorectal cancer, single nucleotide variants, gut microbiota, diagnostic markers

## Abstract

Current metagenomic species-based colorectal cancer (CRC) microbial biomarkers may confuse diagnosis because the genetic content of different microbial strains, even those belonging to the same species, may differ from 5% to 30%. Here, a total of 7549 non-redundant single nucleotide variants (SNVs) were annotated in 25 species from 3 CRC cohorts (n = 249). Then, 22 microbial SNV markers that contributed to distinguishing subjects with CRC from healthy subjects were identified by the random forest algorithm to construct a novel CRC predictive model. Excitingly, the predictive model showed high accuracy both in the training (AUC = 75.35%) and validation cohorts (AUC = 73.08%-88.02%). We further explored the specificity of these SNV markers in a broader background by performing a meta-analysis across 4 metabolic disease cohorts. Among these SNV markers, 3 SNVs that were enriched in CRC patients and located in the genomes of *Eubacterium rectale* and *Faecalibacterium prausnitzii* were CRC specific (AUC = 72.51%-94.07%).

## Introduction

Colorectal cancer (CRC) is the most common cancer, both in men (1-lung, 2-prostate, 3-colorectal, 4-pancreatic cancer) and women (1-lung, 2-breast, 3-colorectal, 4-pancreatic cancer)^1^ and the second most common cause of cancer death after lung cancer.^2^ In the last decade, some studies have highlighted the importance of the gut microbiome in CRC. In particular, many bacteria,^3^ including *Fusobacterium nucleatum, Bacteroides fragilis* and *Escherichia coli* and fungi,^1^ including *Malasseziomycetes* and *Candida* are involved in the development of CRC due to pathogenicity and carcinogenicity *via* multiple mechanisms. Accordingly, numerous noninvasive microbial biomarkers based on metagenomic species or functional genes were developed for early-stage CRC diagnosis.^4-7^ However, the genetic content of different microbial strains, even those belonging to the same species, may differ from 5% to 30% or more,^8^ which may confuse the diagnosis and create a barrier to applying species-level biomarkers.

Therefore, analysis of gut microbial single nucleotide variants (SNVs) can provide an in-depth view of CRC pathogenesis. To date, only one study has reported that intestinal *Bacteroides coprocola* has a characteristic distribution of single nucleotide variants in the T2D patient group compared to healthy controls.^9^ Unfortunately, no study has addressed the association between CRC and gut microbiota at the SNV level, and the profile of gut microbial genomic variation in patients suffering from CRC is largely unknown. Here, for the first time, this challenge was addressed by identifying gut microbial SNVs in CRC patients from three discovery cohorts (n = 249). Additionally, we recruited a validation cohort and resampled discovery cohorts to evaluate the accuracy of the CRC predictive model. Finally, four disease cohorts were used to determine the specificity of the CRC SNV markers. Importantly, a new method was established to predict CRC with high accuracy based on SNV signatures of gut microbiota.

## Results

### SNV annotation, construction and verification of CRC classification model

**Figure 1.**
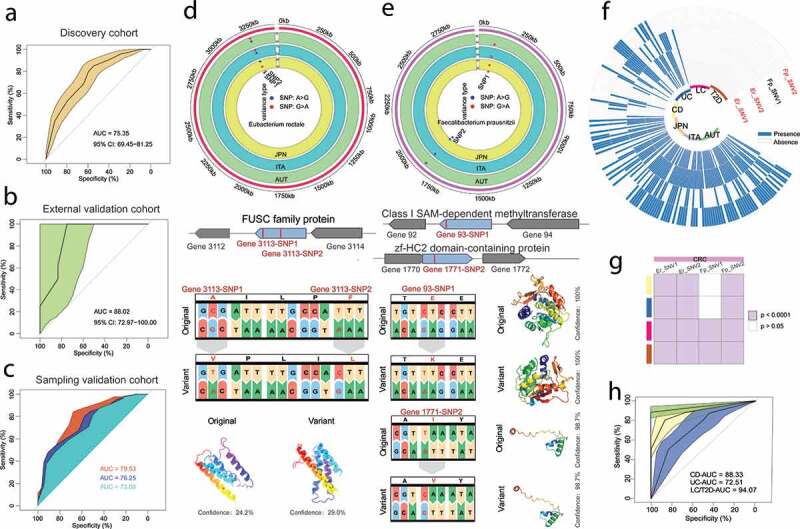
Construction and validation of CRC prediction model based on SNVs. (a) Prediction power in discovery cohorts with the accuracy of 75.35% using 22 SNV markers, including 4 SNVs enriched and 18 SNVs absent in CRC patients. (b) CRC classification accuracy for independent validation set recruited with 8 CRC patients and 12 healthy controls from Hainan Province, China. (c) The performance of the model in resampling validation cohorts. The sampling validation cohorts refer to the random sampling of 100 samples from discovery cohorts. (d) And (e) The related position, functions, amino acid and protein structure and of the four SNVs enriched in CRC. (f) And (g) Heatmaps were used to show the disease specificity of the four SNVs mentioned above, and three SNVs were enriched in CRC cohorts, including Er_SNV1, Er_SNV2 and FP_SNV2. (h) The accuracy of enriched SNVs to classify various diseases, and the accuracy ranged from 72.51% to 94.01%

The depth of the metagenomic sequencing and the coverage of each strain directly affected the discrimination of intestinal microbial SNV identification. Therefore, by using MetaPhlan2 for species annotation, the species with the average relative abundance greater than 0.5% were selected for SNV annotation. The selected reference or representative strains from NCBI and their GenBank accessions are listed in supplemental material 3. In general, 7549 non-redundant SNVs were annotated in 25 species from 249 individuals of 3 cohorts, including Japanese, Australian and Italian individuals. Then, the random forest algorithm was used to find the SNV markers that contributed to distinguishing CRC from healthy controls from three discovery cohorts (mean decrease in accuracy>0). The Wilcoxon rank-sum test was performed to determine the significantly different SNV markers shared among the three cohorts. Finally, a total of 22 SNV markers (including 4 SNVs enriched and 18 SNVs absent in CRC patients) were used to build the prediction model (supplemental materials 5A). The 22 SNVs belong to 4 species, among which 11 belong to *Eubacterium rectale*, nine belong to *Faecalibacterium prausnitzii*, and both *Bifidobacterium pseudocatenulatum* and *Bacteroides vulgatus* contain 1 SNV (supplemental materials 5A). Based on the presence profile of the 22 SNVs (supplemental material 2A), a novel CRC predictive model was constructed. The results showed that the predictive model had a high accuracy of 75.35% in the discovery cohorts (Figure 1a). To further evaluate the robustness of the model, 8 CRC patients and 12 healthy controls were recruited from Hainan Province, China. Our model achieved an area under the receiver-operating curve (AUC) of 88.02% for the validation samples (supplemental material 2B) (Figure 1b). In addition, we resampled to verify the robustness of the model again. We obtained AUCs of 79.53%, 76.25%, and 73.08%, respectively, for the three resampling processes (Figure 1c).

### Functional annotations related to four enriched SNVs and cross-disease model verification

The 4 SNVs enriched in CRC patients were located in the genomes of *Eubacterium rectale* and *Faecalibacterium prausnitzii*. Interestingly, we did not observe any significant difference in the relative abundance of *Eubacterium rectale* and *Faecalibacterium prausnitzii* in all cohorts (supplemental materials 5B, C), which further underlined the sensitivity of gut microbial SNV biomarkers. To gain insights into the 4 enriched SNV functions in CRC, their respective functions were demonstrated according to the feature table from the public database. Further, we observed that all four SNVs were non-synonymous mutations and we predicted the proteins structure of the related genes using Phyre2^10^ (Figure 1d,e). The 2 SNVs located in the *Eubacterium rectale* genome were related to the function of fusaric acid resistance protein-like (FUSC family protein, WP_012744219.1). The other two CRC-enriched SNVs in *Faecalibacterium prausnitzii* were assigned to the function of methyltransferase and ZF-HC2 domain-containing protein. To further explore the specificity of the 4 CRC-enriched SNVs in a broader background, a meta-analysis was performed across four metabolic disease cohorts, including ulcerative colitis (UC), Crohn’s disease (CD), liver cirrhosis (LC), and type 2 diabetes (T2D). The results showed that 3 of 4 SNVs were disease specific. In contrast, the SNV1 of *Faecalibacterium prausnitzii* showed no difference between CRC and intestinal IBD diseases, including CD and UC (Figure 1f,g). Subsequently, the three SNVs were used to distinguish CRC from other conditions, and the accuracy ranged from 72.51% to 94.01%, which implied the outstanding specificity of the 3 SNV markers in CRC disease (supplemental material 2 C) (Figure 1h).

## Discussion

According to the above findings at the intestinal microbial SNV level, there are many potential applications worth discussing that are not limited to the development of potential drug targets reported by a previous study.^9^ Notably, fusaric acid (FA) is a ubiquitous but neglected fungal toxin.^11^ So, it should be emphasized that the human gut microbiota consists of not only bacteria but also viruses, fungi, and Archaea . Possible changes in FA resistance should be taken seriously in patients with CRC due to two significantly enriched SNVs related to FUSC family proteins. Interestingly, FA is a causative agent of esophageal cancer^12^ and decreases p53 expression;^13^ on the other hand, FA has activity against head and neck squamous cell carcinoma^14,15^ and human esophageal epithelial carcinoma cells.^16^ Therefore, the SNV profiles indicate that the effect of FA on human colorectal cancer cells should be further investigated. In summary, our study suggests that SNV distributions should be further examined to determine, in-depth, potential changes in the function of the gut microbiota. Even so, verification with larger and more regional cohorts still needs to be carried out, and the isolation and confirmation of strains is especially critical. The Study have summarized gut microbes associated with CRC development,^3^ including *Fusobacterium nucleatum, Bacteroides fragilis, Escherichia col*i, *Enterococcus faecalis, Helicobacter hepaticus, Peptostreptococcus anaerobius, Helicobacter pylori, Streptococcus bovis*, and *Porphyromonas gingivalis*. However, *Eubacterium rectale* and *Faecalibacterium prausnitzii* have not received enough attentions in patients suffering CRC. Only few studies implied *Faecalibacterium prausnitzii* as the potential probiotic because of its maintenance function of gut homeostasis.^3,17^ Interestingly, both *Eubacterium rectale* and *Faecalibacterium prausnitzii* were crucial intestinal microbes for butyrate producing,^18,19^ and butyrate represented SCFAs were considered as microbial metabolites with anti-tumorigenic properties and may contribute to the prevention of CRC.^20,21^ However, no significant difference was observed in the abundance of *Eubacterium rectale* and *Faecalibacterium prausnitzii* in all cohorts, which implied even the microbial relative abundance has not changed, a number of significant functional mutations have occurred in microbial genome which participate in the development of CRC.^22^

The present predictive model constructed by 22 CRC gut microbial SNVs exhibited high accuracy in training and validation cohorts, and the 3 CRC-enriched microbial SNVs were disease specific. Here, we not only explored the potential correlation between gut microbial genomic mutations and CRC disease but also developed a feasible noninvasive CRC diagnostic method. SNVs profiles of bacteria in the gut of CRC patients has been analyzed in this study, whereas it was observed that the CRC stage is strongly related to the fungal microbes.^1^ So, fungal SNVs in the gut of patients with CRC should be analyzed in future studies.

## Materials and methods

### Sequence data collection

Fecal shotgun metagenomic data of human CRC patients and healthy cohorts were collected. For discovery cohorts, raw SRA files and sample information from three studies were downloaded from NCBI using the following accessions: ERP008729 for Austria,^23^ DRA006684 for Japan,^24^ and SRP136711 for Italy.^25^ A total of 118 cases and 131 healthy controls were included in this meta-analysis (Table 1). For external validation cohorts, we recruited 8 CRC patients and 12 healthy controls from Hainan Province, China. The patient recruitment and sequencing pipeline can be found in supplemental materials 1. The sequence data have been deposited in the NCBI database under PRJNA663646. We also randomly sampled the discovery cohorts using Rstudio, performing the sampling for a total of three times; each time, 100 individuals were used to evaluate the prediction model, and the random sampling process is shown in supplemental materials 2. To investigate whether four enriched SNVs found from the CRC cohorts were disease specific, we also collected information for four other common diseases, and thirty samples were randomly selected for each disease (Table 1), including inflammatory bowel diseases (IBDs),^26^ type 2 diabetes^27^ and liver cirrhosis^28^ .

**Table 1.** Fecal metagenomic studies included in this meta-analysis

Cohorts	No.of cases	No.of controls	Accession
Discovery cohorts Austria(AUT)	46	63	ERP008729
Italy(ITA)	32	28	SRP136711
Japan(JPN)	40	40	DRA006684
External validation cohorts^#1^			
China(CHN)	8	12	PRJNA663646
Sampling validation cohorts^#2^			
Sampling cohort 1	44	56	-
Sampling cohort 2	46	54	-
Sampling cohort 3	48	52	-
Disease comparison cohorts			
IBD-UC	30	-	PRJNA400072
IBD-CD	30	-	PRJNA400072
Type 2 diabetes(T2D)	30	-	PRJNA422434
Liver cirrhosis(LC)	30	-	PRJEB6337

^#1^: The data for external validation cohorts has not been published.

^#2^: The sampling validation cohorts refer to the random sampling of 100 samples from discovery cohorts.

### Identification of microbial taxonomy and SNV calling

Shotgun metagenomic sequencing and quality control information can be found in supplemental materials 1. For metagenomic species annotation, MetaPhlan2 software was applied for taxonomic classification.^29^ We next employed MIDAS (Metagenomic Intra-Species Diversity Analysis System) to profile the species-level SNV frequency and gene contents in the gut microbiota.^30^ Briefly, we constructed reference bacteria in a high-abundance genome database. Information on all 25 reference strains can be found in supplemental material 3. Then, the shotgun metagenomic sequencing reads were mapped to the database for SNV calling. More information on the code can be found in supplemental materials 4 and GitHub: https://github.com/HNUmcc/CRC-SNP.

### Statistics statement

The statistical analyses were conducted using R software. Randomforest test was performed by the “randomForest” package. Further, we selected the differential SNVs shared among three discovered cohorts based on randomforest results using the Wilcoxon rank-sum test (*p* < .05), which were considered to be potential biomarkers. Boxplot was shown by the “ggplot2” package. Receiver operator characteristic (ROC) analysis was used to assess the performance of the microbial biomarkers using the “pROC” package in R. The heatmap was constructed using TBtools software.^31^

## Supplementary Material

Supplemental Material

## Data Availability

The external validation cohort sequence data reported in this paper have been deposited in the NCBI database (metagenomic sequencing data: PRJNA663646). The analysis code for metaphlan2 and SNV calling can be found in supplemental materials 4 and had been deposited in Github: https://github.com/HNUmcc/CRC-SNP.
