# Supplementary material for: Establishing a novel colorectal cancer predictive model based on unique gut microbial single nucleotide variant markers
Source: Gut Microbes. 2021 Jan 11;13(1):1869505. doi: 10.1080/19490976.2020.1869505 (PMC7808391; doi:10.1080/19490976.2020.1869505)
Supplement: Supplemental Material [file KGMI_A_1869505_SM9827.zip › SUPPLEMENT/Supplemental material 1.docx]

**Supplemental material 1:**

**Hainan patient cohort recruitment (External validation cohort)**

The participants from Hainan, China in the study were informed about the study and were provided with written consent. The protocol for the study was approved by the Ethics Committee Hainan University.

Fecal samples were collected from 8 individuals diagnosed with CRC and 12 healthy individuals who underwent standard colonoscopy screening at Haikou people's Hospital, Hainan, China. Healthy cases were disease-free and had no antibiotics or probiotics for nearly three months. Sampling and all described subsequent steps were conducted per the approved guidelines. Fecal samples were collected from each subject in the morning before the first meal. Samples were stored at -40°C after collection and were transported to the research lab within 2 hours. All the CRC diagnosed individuals were male between 38 and 77 years of age and body mass index (BMI) of 21.25±1.64, and there were no significant difference between healthy controsl and CRC. DNA extraction was carried out using CWBIO Stool Genomic DNA Kit (CW2092, CWBIO, China) as per the manufacturer’s guidelines.

**Shotgun metagenomic sequencing and quality control**

For Hainan cohort, whole-genome shotgun sequencing of the samples was carried out using Illumina HiSeq 2500 instrument. Libraries were generated using a fragment length of approximately 300 bp. Paired-end reads were created using 150 bp in the forward and reverse directions. The reads were trimmed using Sickle and were subsequently aligned to the human genome with the reference genome (hg38 database) to remove the host DNA fragments.

**Supplemental material 2:**

The random sampling process

| sample | ID | group | sample | ID | group | sample | ID | group |
| --- | --- | --- | --- | --- | --- | --- | --- | --- |
| ITA_C1 | 1 | CRC | JPN_C24 | 84 | CRC | AUT_C27 | 167 | CRC |
| ITA_C2 | 2 | CRC | JPN_C25 | 85 | CRC | AUT_C28 | 168 | CRC |
| ITA_C3 | 3 | CRC | JPN_C26 | 86 | CRC | AUT_C29 | 169 | CRC |
| ITA_C4 | 4 | CRC | JPN_C27 | 87 | CRC | AUT_C30 | 170 | CRC |
| ITA_C5 | 5 | CRC | JPN_C28 | 88 | CRC | AUT_C31 | 171 | CRC |
| ITA_C6 | 6 | CRC | JPN_C29 | 89 | CRC | AUT_C32 | 172 | CRC |
| ITA_C7 | 7 | CRC | JPN_C30 | 90 | CRC | AUT_C33 | 173 | CRC |
| ITA_C8 | 8 | CRC | JPN_C31 | 91 | CRC | AUT_C34 | 174 | CRC |
| ITA_C9 | 9 | CRC | JPN_C32 | 92 | CRC | AUT_C35 | 175 | CRC |
| ITA_C10 | 10 | CRC | JPN_C33 | 93 | CRC | AUT_C36 | 176 | CRC |
| ITA_C11 | 11 | CRC | JPN_C34 | 94 | CRC | AUT_C37 | 177 | CRC |
| ITA_C12 | 12 | CRC | JPN_C35 | 95 | CRC | AUT_C38 | 178 | CRC |
| ITA_C13 | 13 | CRC | JPN_C36 | 96 | CRC | AUT_C39 | 179 | CRC |
| ITA_C14 | 14 | CRC | JPN_C37 | 97 | CRC | AUT_C40 | 180 | CRC |
| ITA_C15 | 15 | CRC | JPN_C38 | 98 | CRC | AUT_C41 | 181 | CRC |
| ITA_C16 | 16 | CRC | JPN_C39 | 99 | CRC | AUT_C42 | 182 | CRC |
| ITA_C17 | 17 | CRC | JPN_C40 | 100 | CRC | AUT_C43 | 183 | CRC |
| ITA_C18 | 18 | CRC | JPN_H1 | 101 | Con | AUT_C44 | 184 | CRC |
| ITA_C19 | 19 | CRC | JPN_H2 | 102 | Con | AUT_C45 | 185 | CRC |
| ITA_C20 | 20 | CRC | JPN_H3 | 103 | Con | AUT_C46 | 186 | CRC |
| ITA_C21 | 21 | CRC | JPN_H4 | 104 | Con | AUT_H1 | 187 | Con |
| ITA_C22 | 22 | CRC | JPN_H5 | 105 | Con | AUT_H2 | 188 | Con |
| ITA_C23 | 23 | CRC | JPN_H6 | 106 | Con | AUT_H3 | 189 | Con |
| ITA_C24 | 24 | CRC | JPN_H7 | 107 | Con | AUT_H4 | 190 | Con |
| ITA_C25 | 25 | CRC | JPN_H8 | 108 | Con | AUT_H5 | 191 | Con |
| ITA_C26 | 26 | CRC | JPN_H9 | 109 | Con | AUT_H6 | 192 | Con |
| ITA_C27 | 27 | CRC | JPN_H10 | 110 | Con | AUT_H7 | 193 | Con |
| ITA_C28 | 28 | CRC | JPN_H11 | 111 | Con | AUT_H8 | 194 | Con |
| ITA_C29 | 29 | CRC | JPN_H12 | 112 | Con | AUT_H9 | 195 | Con |
| ITA_C30 | 30 | CRC | JPN_H13 | 113 | Con | AUT_H10 | 196 | Con |
| ITA_C31 | 31 | CRC | JPN_H14 | 114 | Con | AUT_H11 | 197 | Con |
| ITA_C32 | 32 | CRC | JPN_H15 | 115 | Con | AUT_H12 | 198 | Con |
| ITA_H1 | 33 | Con | JPN_H16 | 116 | Con | AUT_H13 | 199 | Con |
| ITA_H2 | 34 | Con | JPN_H17 | 117 | Con | AUT_H14 | 200 | Con |
| ITA_H3 | 35 | Con | JPN_H18 | 118 | Con | AUT_H15 | 201 | Con |
| ITA_H4 | 36 | Con | JPN_H19 | 119 | Con | AUT_H16 | 202 | Con |
| ITA_H5 | 37 | Con | JPN_H20 | 120 | Con | AUT_H17 | 203 | Con |
| ITA_H6 | 38 | Con | JPN_H21 | 121 | Con | AUT_H18 | 204 | Con |
| ITA_H7 | 39 | Con | JPN_H22 | 122 | Con | AUT_H19 | 205 | Con |
| ITA_H8 | 40 | Con | JPN_H23 | 123 | Con | AUT_H20 | 206 | Con |
| ITA_H9 | 41 | Con | JPN_H24 | 124 | Con | AUT_H21 | 207 | Con |
| ITA_H10 | 42 | Con | JPN_H25 | 125 | Con | AUT_H22 | 208 | Con |
| ITA_H11 | 43 | Con | JPN_H26 | 126 | Con | AUT_H23 | 209 | Con |
| ITA_H12 | 44 | Con | JPN_H27 | 127 | Con | AUT_H24 | 210 | Con |
| ITA_H13 | 45 | Con | JPN_H28 | 128 | Con | AUT_H25 | 211 | Con |
| ITA_H14 | 46 | Con | JPN_H29 | 129 | Con | AUT_H26 | 212 | Con |
| ITA_H15 | 47 | Con | JPN_H30 | 130 | Con | AUT_H27 | 213 | Con |
| ITA_H16 | 48 | Con | JPN_H31 | 131 | Con | AUT_H28 | 214 | Con |
| ITA_H17 | 49 | Con | JPN_H32 | 132 | Con | AUT_H29 | 215 | Con |
| ITA_H18 | 50 | Con | JPN_H33 | 133 | Con | AUT_H30 | 216 | Con |
| ITA_H19 | 51 | Con | JPN_H34 | 134 | Con | AUT_H31 | 217 | Con |
| ITA_H20 | 52 | Con | JPN_H35 | 135 | Con | AUT_H32 | 218 | Con |
| ITA_H21 | 53 | Con | JPN_H36 | 136 | Con | AUT_H33 | 219 | Con |
| ITA_H22 | 54 | Con | JPN_H37 | 137 | Con | AUT_H34 | 220 | Con |
| ITA_H23 | 55 | Con | JPN_H38 | 138 | Con | AUT_H35 | 221 | Con |
| ITA_H24 | 56 | Con | JPN_H39 | 139 | Con | AUT_H36 | 222 | Con |
| ITA_H25 | 57 | Con | JPN_H40 | 140 | Con | AUT_H37 | 223 | Con |
| ITA_H26 | 58 | Con | AUT_C1 | 141 | CRC | AUT_H38 | 224 | Con |
| ITA_H27 | 59 | Con | AUT_C2 | 142 | CRC | AUT_H39 | 225 | Con |
| ITA_H28 | 60 | Con | AUT_C3 | 143 | CRC | AUT_H40 | 226 | Con |
| JPN_C1 | 61 | CRC | AUT_C4 | 144 | CRC | AUT_H41 | 227 | Con |
| JPN_C2 | 62 | CRC | AUT_C5 | 145 | CRC | AUT_H42 | 228 | Con |
| JPN_C3 | 63 | CRC | AUT_C6 | 146 | CRC | AUT_H43 | 229 | Con |
| JPN_C4 | 64 | CRC | AUT_C7 | 147 | CRC | AUT_H44 | 230 | Con |
| JPN_C5 | 65 | CRC | AUT_C8 | 148 | CRC | AUT_H45 | 231 | Con |
| JPN_C6 | 66 | CRC | AUT_C9 | 149 | CRC | AUT_H46 | 232 | Con |
| JPN_C7 | 67 | CRC | AUT_C10 | 150 | CRC | AUT_H47 | 233 | Con |
| JPN_C8 | 68 | CRC | AUT_C11 | 151 | CRC | AUT_H48 | 234 | Con |
| JPN_C9 | 69 | CRC | AUT_C12 | 152 | CRC | AUT_H49 | 235 | Con |
| JPN_C10 | 70 | CRC | AUT_C13 | 153 | CRC | AUT_H50 | 236 | Con |
| JPN_C11 | 71 | CRC | AUT_C14 | 154 | CRC | AUT_H51 | 237 | Con |
| JPN_C12 | 72 | CRC | AUT_C15 | 155 | CRC | AUT_H52 | 238 | Con |
| JPN_C13 | 73 | CRC | AUT_C16 | 156 | CRC | AUT_H53 | 239 | Con |
| JPN_C14 | 74 | CRC | AUT_C17 | 157 | CRC | AUT_H54 | 240 | Con |
| JPN_C15 | 75 | CRC | AUT_C18 | 158 | CRC | AUT_H55 | 241 | Con |
| JPN_C16 | 76 | CRC | AUT_C19 | 159 | CRC | AUT_H56 | 242 | Con |
| JPN_C17 | 77 | CRC | AUT_C20 | 160 | CRC | AUT_H57 | 243 | Con |
| JPN_C18 | 78 | CRC | AUT_C21 | 161 | CRC | AUT_H58 | 244 | Con |
| JPN_C19 | 79 | CRC | AUT_C22 | 162 | CRC | AUT_H59 | 245 | Con |
| JPN_C20 | 80 | CRC | AUT_C23 | 163 | CRC | AUT_H60 | 246 | Con |
| JPN_C21 | 81 | CRC | AUT_C24 | 164 | CRC | AUT_H61 | 247 | Con |
| JPN_C22 | 82 | CRC | AUT_C25 | 165 | CRC | AUT_H62 | 248 | Con |
| JPN_C23 | 83 | CRC | AUT_C26 | 166 | CRC | AUT_H63 | 249 | Con |


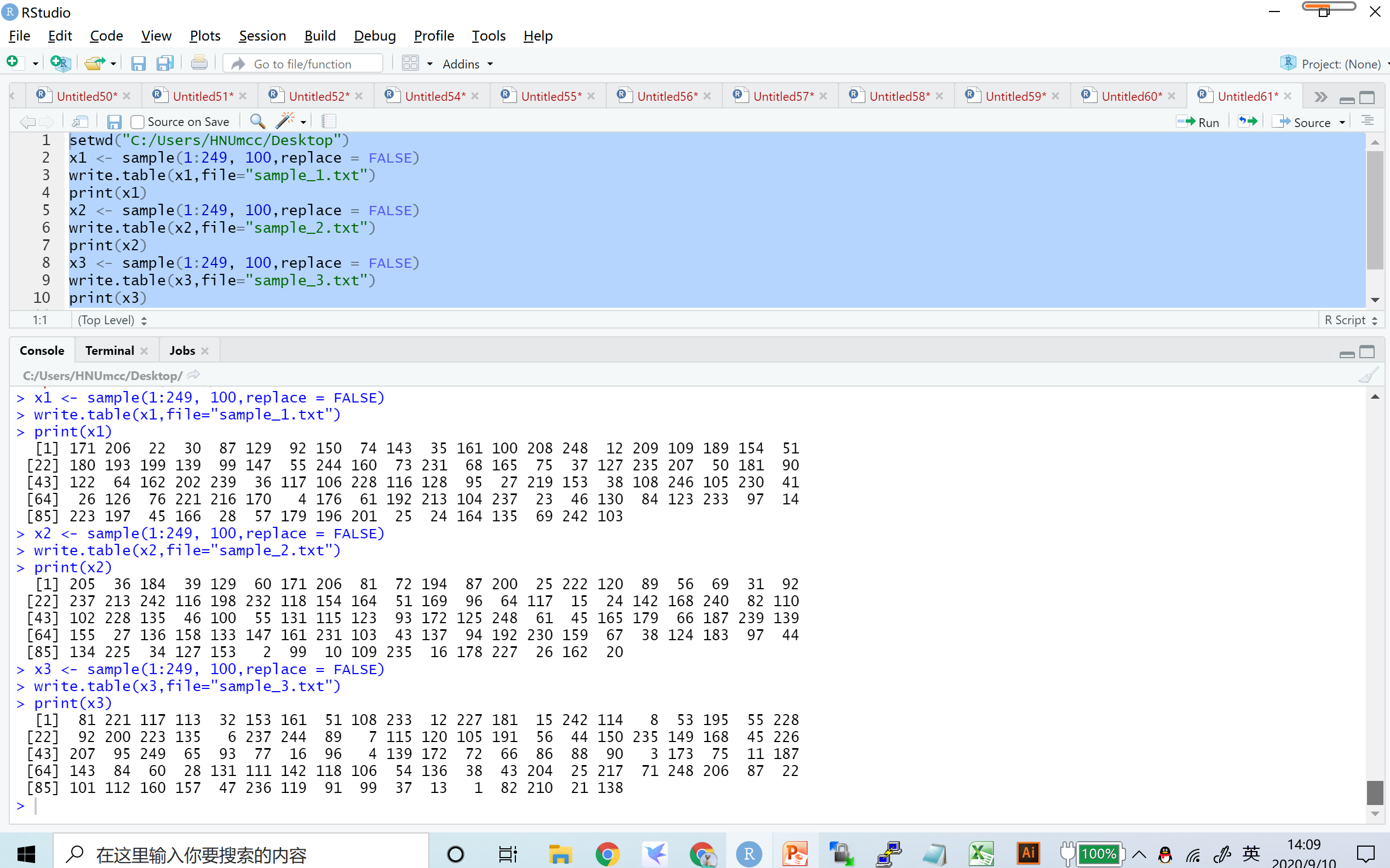


**Supplemental material 3:**

25 reference strains information

| Species | Strains | Accession |
| --- | --- | --- |
| *Faecalibacterium prausnitzii* | *Faecalibacterium prausnitzii* ATCC 27768 | GCA_003312465.1 |
| *Eubacterium rectale* | *Eubacterium rectale* ATCC 33656 | GCA_000020605.1 |
| *Prevotella copri* | *Prevotella copri* DSM 18205 | GCA_000157935.1 |
| *Bifidobacterium adolescentis* | *Bifidobacterium adolescentis* ATCC 15703 | GCA_000010425.1 |
| *Bifidobacterium longum* | *Bifidobacterium longum* NCC2705 | GCA_000007525.1 |
| *Bacteroides vulgatus* | *Bacteroides vulgatus* ATCC 8482 | GCA_000012825.1 |
| *Akkermansia muciniphila* | *Akkermansia muciniphila* ATCC BAA-835 | GCA_000020225.1 |
| *Bacteroides uniformis* | *Bacteroides uniformis* ATCC 8492 | GCA_000154205.1 |
| *Bacteroides stercoris* | *Bacteroides stercoris* ATCC 43183 | GCA_000154525.1 |
| *Bacteroides dorei* | *Phocaeicola dorei* | GCA_902387545.1 |
| *Bacteroides ovatus* | *Bacteroides ovatus* | GCA_001314995.1 |
| *Bacteroides massiliensis* | *Bacteroides massiliensis* DSM 17679 | GCA_000382445.1 |
| *Bacteroides caccae* | *Bacteroides caccae* | GCA_001405955.1 |
| *Bacteroides coprocola* | *Bacteroides coprocola* DSM 17136 | GCA_000154845.1 |
| *Bifidobacterium bifidum* | *Bifidobacterium bifidum* PRL2010 | GCA_000165905.1 |
| *Bacteroides thetaiotaomicron* | *Bacteroides thetaiotaomicron* VPI-5482 | GCA_000011065.1 |
| *Bacteroides fragilis* | *Bacteroides fragilis* YCH46 | GCA_000009925.1 |
| *Bacteroides plebeius* | *Bacteroides plebeius* DSM 17135 | GCA_000187895.1 |
| *Bifidobacterium pseudocatenulatum* | *Bifidobacterium pseudocatenulatum* | GCA_003952825.1 |
| *Bacteroides eggerthii* | *Bacteroides eggerthii* | GCA_900445565.1 |
| *Fusobacterium nucleatum* | *Fusobacterium nucleatum* subsp. nucleatum ATCC 25586 | GCA_000007325.1 |
| *Gemella morbillorum* | *Gemella morbillorum* | GCA_009730315.1 |
| *Parvimonas micra* | *Parvimonas micra* | GCA_000800295.1 |
| *Peptostreptococcus stomatis* | *Peptostreptococcus stomatis* DSM 17678 | GCA_000147675.2 |
| *Solobacterium moorei* | *Solobacterium moorei* DSM 22971 | GCA_000425005.1 |

**Supplemental material 4:**

#For metagenomic data taxonomy annotation

Software: MetaPhlan2

python metaphlan2.py h1.fastq --input_type fastq > h1.taxa.txt

python merge_metaphlan_tables.py *taxa.txt taxa_all.txt

# For SNV calling

Software: bowtie2, bcftools

bowtie2 -p 12 -x ref.index --no-mixed --very-sensitive --n-ceil 0,0.01 -1 h1.fq | samtools sort -O bam -@ 24 -o - > h1ref.bam

bcftools mpileup -C 50 -Ou -m 3 -F 0.0002 -f ref.fa h1ref.bam > h1ref.bcf

bcftools call -c --variants-only -Ob --ploidy 1 h1ref.bcf > h1.bcf

bcftools view -Ov h1.bcf | vcfutils.pl varFilter -d 100 > h1.vcf

**Supplemental material 4:**


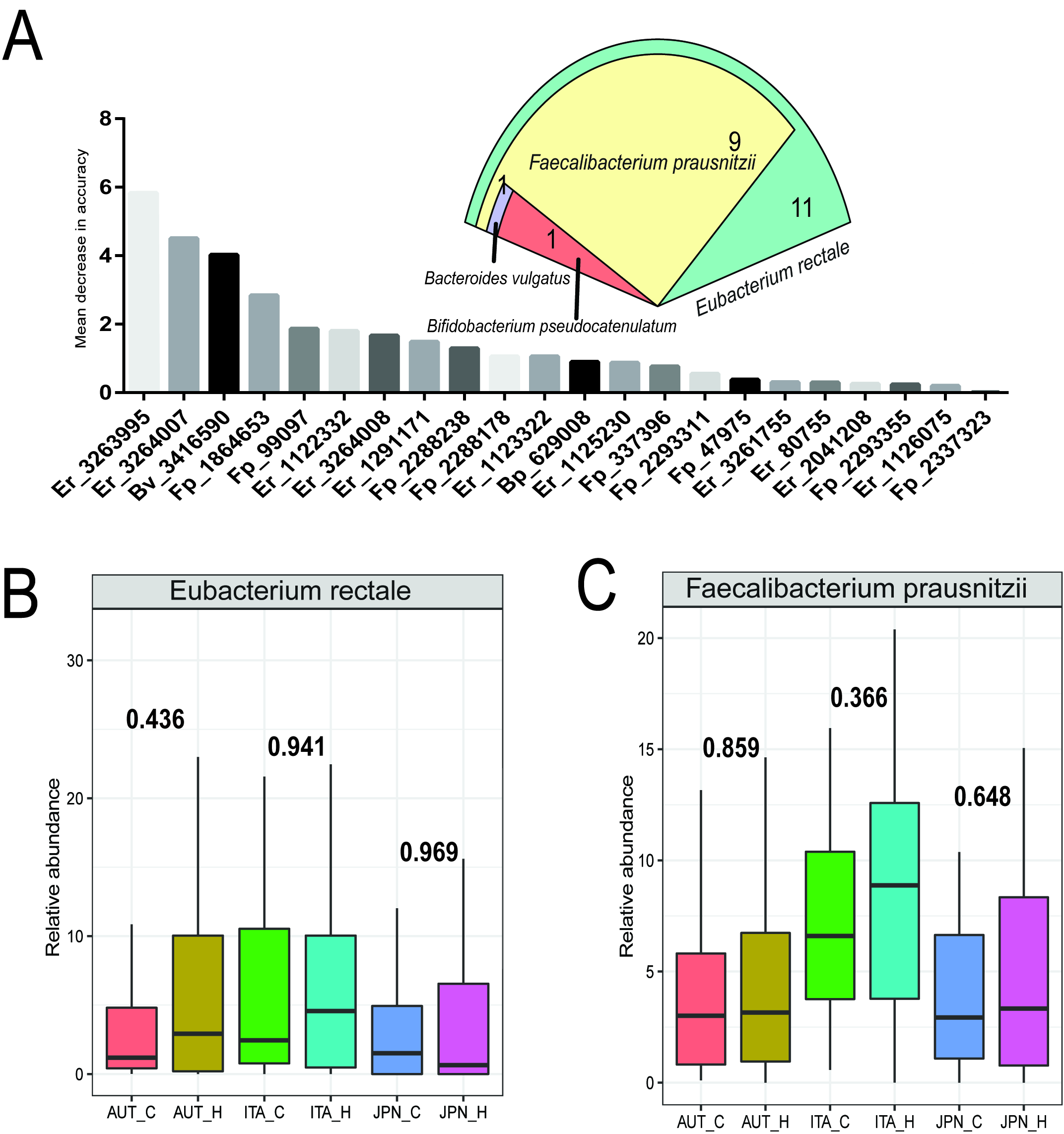


**Figure 1 Target species and SNVs. (A)** RandomForest results of 22 SNVs in discovery cohorts. **(B) And (C)** The relative abundance of *Eubacterium rectale* and *Faecalibacterium prausnitzii* in all discovery cohorts
